# Improved Morphine-Loaded Hydrogels for Wound-Related Pain Relief

**DOI:** 10.3390/pharmaceutics11020076

**Published:** 2019-02-12

**Authors:** Dinis Mateus, Joana Marto, Patrícia Trindade, Humberto Gonçalves, Ana Salgado, Paula Machado, António Melo-Gouveia, Helena M. Ribeiro, António J. Almeida

**Affiliations:** 1 Serviço Farmacêutico, Instituto Português de Oncologia de Lisboa, Francisco Gentil, Lisboa, Portugal; dinis_mateus@hotmail.com (D.M.); ptrindade@ipolisboa.min-saude.pt (P.T.); hgoncalves@ipolisboa.min-saude.pt (H.G.); agouveia@ipolisboa.min-saude.pt (A.M.-G.); 2 Research Medicine Research Institute (iMed.ULisboa), Faculty of Pharmacy, Universidade de Lisboa, Lisboa, Portugal; jmmarto@ff.ulisboa.pt (J.M.); asalgado@ff.ulisboa.pt (A.S.); hribeiro@campus.ul.pt (H.M.R.); 3 Laboratório de Controlo Microbiológico, ADEIM, Faculty of Pharmacy, Universidade de Lisboa, Lisboa, Portugal; paula_v_machado@hotmail.com

**Keywords:** painful wounds, hydrogels, skin ulcers, morphine, topical opioids, quality risk management

## Abstract

The use of morphine applied topically to painful wounds has potential advantages, such as dose reduction, fewer side effects and compound formulations, have been proposed for this purpose. Given the potential high impact of drug product quality on a patient’s health, the aim of the present study was to develop two stable sterile hydrogels containing morphine hydrochloride, intended for topical application on painful wounds. Two carboxymethylcellulose sodium-based hydrogels were prepared containing 0.125% *w*/*w* (F1-MH semi-solid formulation) and 1.0% *w*/*w* (F2-MH fluid formulation) morphine hydrochloride (MH), respectively. Studies included a risk assessment approach for definition of the quality target product profile (QTPP) and assessment of critical quality attributes (CQA) of the hydrogels to support product quality and safety. Safe, odourless, yellowish, translucent and homogeneous gels were obtained, with suitable microbiological and pharmaceutical characteristics. The active substance concentration was adapted according to the characteristics of the dose-metering device. Release profiles were investigated using Franz diffusion cells, and characterised by different kinetic models. Increasing gel viscosity prolonged drug release, with rates of 17.9 ± 2.2 μg·cm^−2^·h^−1^ (F1-MH) and 258.0 ± 30.4 μg·cm^−2^·h^−1^ (F2-MH), allowing for the reduction of the number of applications and improving patient compliance. The gels proved to be stable for up to 60 days at room temperature. The semi-solid and fluid MH-containing hydrogel formulations are safe, stable and suitable for use in hospital settings, which is rather important for wound-related pain management in cancer palliative care or burn patients.

## 1. Introduction

Wound management is a therapeutic area of increasing interest that involves healing and palliative care of wound-related pain and symptoms [1]. The latter can be difficult to treat, being only partially responsive to conventional systemic analgesics [2]. Scientific evidence supports pain relief after topical application of some classes of drugs with analgesic potential, such as nonsteroidal anti-inflammatory drugs, opioids, local anaesthetics, cannabinoids, cholinergic receptor agonists, etc. [3,4,5]. There are two main reasons for selecting the topical route of administration to obtain pain relief. The first is the therapeutic intent of maximising the drug concentration at specific target tissues in order to increase the effect, while minimising drug concentration in other more distant sites, thus reducing side effects. Secondly, patients like the concept of applying a medication on the spot where they feel pain [6]. Concerning pain monitoring in cancer patients, elderly, chronically ill and/or polymedicated patients, the issue of adverse effects of medications and the high propensity for drug interaction is of particular importance. Furthermore, topically applied analgesics have the possibility of achieving pain relief while setting aside the need to consider alternatives that may be more toxic [3].

Interest in using topically applied morphine on painful ulcers has been increasing, particularly for pain associated with pressure ulcers [3,7], or that resulting from radiation-induced dermatitis and tumor infiltration [8]. The subject is still controversial [9], but preclinical studies indicate that morphine has an analgesic effect when administered topically, which is supported by direct scientific evidence from properly designed and implemented clinical studies [3,6,9,10]. The presence of opioid receptors in the skin has long been reported as part of the endogenous opioid system, mediating the effects of opioid peptides (i.e. enkephalins, endorphins, dynorphins and endomorphins), as well as of exogenous opiate alkaloids, such as morphine [11]. Opioid receptors are found in peripheral nerve terminals, which are similar to those located in the central nervous system. In normal circumstances, opioid receptors are not evident in common tissues, but they can become detectable, within minutes or hours, after the onset of an inflammatory process [10]. Pain relief occurs via inhibition of sensory neurons and the release of pro-inflammatory neuropeptides [9]. Thus, the application of small doses of opioids for significant analgesia is possible, also reducing systemic absorption. As a result, patients can achieve a superior analgesia, requiring less medication and increasing treatment compliance, while reducing or eliminating the risk of related side effects [4,12].

Over the last decades, the use of extemporaneously prepared morphine-containing topical formulations has been proposed for topical treatment of painful wounds [7,9,10,12]. Morphine has some advantages over other opioids, such as low cost, and relatively easy accessibility to sterile injectable liquid preparations that can be incorporated in formulations, such as hydrogels, with different consistencies: from fluids to semi solid [12]. 

Pharmaceutical hydrogels have proven to be excellent tools in the treatment of wounds because of their beneficial healing characteristics [13]. They are formed by the combination of one or more hydrophilic polymers selected for application to wounds and burns according to their capacity to absorb more than their weight in water and film formation, reducing the potential for irritation when in contact with tissue [14]. Their advantages with respect to application to wounds and burns include: high bioadhesion to the wound surface, moisture and vapor permeability necessary for healing of the injured area [15], infection control and wound healing, due to anti-bacterial action via attaching to microbes and removing them from wound [16].

However, most compounded preparations described in the literature consist of incorporating morphine in a commercial gel formulation (IntraSite® Gel, Smith & Nephew, London, UK) and few make reference to preparation techniques validated to ensure sterility of the final product, since semi-solid formulations intended for cutaneous application on severely injured skin must be sterile [15,17,18]. In addition, scarce data on the physicochemical and microbiological properties and stability of these preparations can be found in the literature [17]. This is rather important in pharmaceutical compounding, because hospital pharmacists frequently have to formulate medicines for patients with specific unmet medical needs, such as wound-related pain management in cancer palliative care or burn patients, for whom wound care is painful, and the ability to provide pain and anxiety relief may be a limiting step for wound management [19].

The aim of the present study was to develop two stable sterile hydrogels containing morphine, intended to treat painful wounds: semi-solid and fluid formulations suitable for spreading or spraying over with severe painful wounds. To prioritise the criticality of possible quality risk, studies included a risk assessment approach for careful excipient selection, definition of the quality target product profile (QTPP), assessment of critical quality attributes (CQA) of the hydrogels, as well as their pharmaceutical performance.

## 2. Material and Methods

### 2.1. Materials

Morphine FHC® 20 mg·mL^−1^ (aqueous injectable solution) was purchased from FHC Farmacêutica, Lda (Mortágua, Portugal) and used as source of morphine hydrocloride (MH). Carboxymethylcellulose sodium (NaCMC; high viscosity: 700–1500 mPa·S) was acquired from Sigma Aldrich (Darmstadt, Germany). Methylparaben and propylparaben were obtained from Fagron Iberica (Barcelona, Spain). Glycerol was purchased from Vencilab (Vila Nova de Gaia, Portugal) and sterile water for injections was obtained from B. Braun Medical, Lda. (Queluz de Baixo, Portugal). IntraSite® Gel (Smith & Nephew, London, UK) and Varihesive® Hydrogel (Convatec, Reading, UK) were also used for comparison purposes. Acetonitrile HPLC-gradient grade (Panreac, Castellar del Vallès, Spain) and ethanol (Merck, Darmstadt, Germany) were also used. Tuffryn® membranes (polisulfone membrane disc filters, 0.45 µm) were purchased from the Pall Corporation (East Hills, NY, USA). All other ingredients used were of analytical grade or equivalent.

### 2.2. Methods 

#### 2.2.1. Identification of QTPP and CQAs

The quality attributes of the product were defined based on its desired quality profile, according to the guideline ICH Q8(R2) [20]. The QTPPs vary according to scientific, regulatory and practical considerations and limitations. The most important characteristics of the morphine hydrogels for treating painful wounds were: route of administration, dosage form, strength, the CQAs and stability (Table 1) [21].

#### 2.2.2. Risk Analysis of CQAs

The first step in the risk assessment was to collect all the possible factors that could impact product quality (ICH Q9) [25]. So, the potential variables, which could influence the desired quality attributes, were identified and classified using an Ishikawa diagram. It allowed for prioritising the possible risk factors associated to hydrogel stability, as well as the process parameters. Assay, pH, viscosity, efficacy of antimicrobial preservation and sterility were identified as CQAs.

Among these, sterility is a crucial attribute. According to the United States Pharmacopeia (USP) <797> Pharmaceutical Compounding—Sterile Preparations, the herein described MH-containing hydrogels may be classified as medium-risk level preparations [23]. Therefore, it was crucial to know the general microbial hazards involved in a drug product’s life cycle and define what actions could be taken to prevent contamination. Relying only on finished product testing is not adequate for controlling microbiological contaminations and, for this reason, a safety plan was carefully prepared to assure sterility of the final products [26]. So, the following critical procedures were pointed out, as well as the associate risk and preventive measures adopted: raw materials selection, sterilisation (gel autoclaving and aseptic compounding for MH incorporation), fractioning and final packaging.

#### 2.2.3. Hydrogels’ Preparation

The stock semi-solid (F1) and fluid (F2) hydrogels were prepared at room temperature by dispersing the aqueous thickening agent NaCMC in water for injections containing methylparaben, propylparaben and glycerol, using magnetic stirring at 400 rpm for 24 h, according to the composition described in Table 2. The stock gels were sterilised by autoclaving at 121 °C for 15 min. Then, the morphine-containing gels (F1-MH and F2-MH) were prepared according to the following procedures. Semi-solid gel F1-MH: 5 mL of morphine hydrochloride aqueous injectable solution 20 mgmL-1 (Morphine FHC®) were added to 80 g of F1 gel, to obtain a preparation with 0.125% (*w*/*w*) final morphine concentration. Fluid gel F2-MH: 50 mL of morphine hydrochloride aqueous injectable solution 20 mg/mL (Morphine FHC®) were added to 50 g of F2 gel to obtain a preparation with 1.0% (*w*/*w*) final morphine of concentration.

**Figure 1:**
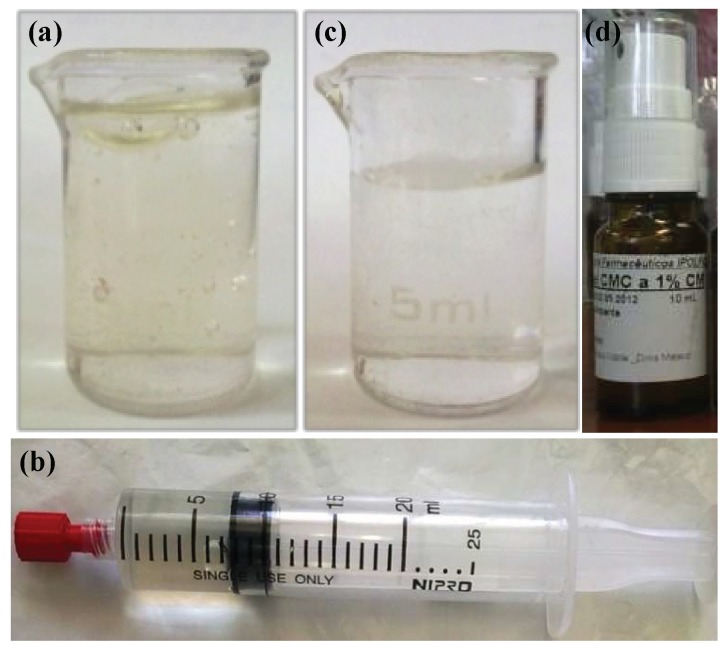
General appearance of the hydrogel formulations: (**a**) Morphine Hydrochloride-containing semi-solid gel (F1-MH) after preparation; (**b**) F1-MH gel packaged in a sterile polypropylene unidose syringe with a luer-lock tip; (**c**) Morphine Hydrochloride-containing fluid gel (F2-MH) after preparation; (**d**) F2-MH gel packaged in a 10 mL amber Type II glass flask with a spray actuator.

After production, the F1-MH gel (Figure 1a) was packaged in sterile 20 mL polypropylene unidose syringes provided with luer lock tips and polypropylene stoppers (Figure 1b), while the F2-MH gel (Figure 1c) was packaged in 10 mL amber Type II glass flasks with a polypropylene stopper assembled with a spray actuator (Figure 1d).

All compounding operations for F1-MH and F2-MH were carried out according to the Pharmaceutical Inspection Convention—Pharmaceutical Inspection Co-operation Scheme (PIC/S) Guide to good practices for the preparation of medicinal products in healthcare establishments, at the Hospital Pharmacy of Instituto Português de Oncologia de Lisboa de Francisco Gentil, in a class A horizontal laminar flow hood (Faster® S.r.l., Italy), inside a class B cleanroom, by qualified personnel [27]. 

#### 2.2.4. Physicochemical Characterisation

The pH of the hydrogels was determined in triplicate for each formulation, after preparation and throughout stability studies, using a potentiometric method (Metrohm® pH Meter 744, glass electrode, Herisau, Switzerland).

The density at 20 °C was determined by weighing, using a 25 mL density bottle for the fluid gel and a 25 ml volumetric flask for the semi-solid gel. Before weighing, the gels were degassed using an ultrasonic bath.

Rheological profiles for all samples were evaluated using a Brookfield Rotation Viscometer® RV DV-II, SSA (Brookfield Engineering Laboratories, Inc., Middleborough, MA, USA), with spindles 7 and 21, at room temperature. The shear rate (s^−1^) versus shear stress (Pa) plots were obtained by submitting the samples to a shear rate sweep from 0.60 to 122 s^−1^, and up and down for 10 min. Apparent viscosity was assessed at two shear rates, i.e., 6.12 s^−1^ for the semi-solid formulations, and 12.24 s^−1^ for the fluid formulations. Determinations were carried out in triplicate for each formulation, both after preparation and throughout stability studies.

#### 2.2.5. Morphine Quantification

Morphine content was determined using a stability-indicating HPLC method described elsewhere [28], slightly modified as follows. A HP 1100 series liquid chromatography System (VWR International, Alfragide, Portugal) was used provided with a pump G1310A, an autosampler G1329A, an UV detector G1328A and the Value Solution ChemStation software. The isocratic method used a reversed-phase column (Lichrospher 100 RP18, 125 mm × 4 mm, 5 µm) and a mobile phase consisting of phosphate buffer (pH 3.5) and acetonitrile (65:35), at a flow-rate of 1.0 mL·min^−1^, with a 10 µL sample injection volume. The auto sampler chamber was maintained at room temperature and the eluted peaks were monitored at 235 nm. The running time was 10 min. The quantification of methylparaben and propylparaben was performed using this same HPLC procedure. The method was validated according to the ICH Q2 (R1) guideline for specificity, linearity, accuracy and precision [29].

#### 2.2.6. Sterility Testing

Semi-solid preparations for cutaneous applications intended for use on severely injured skin must be sterile. The sterility test was performed according to the European Pharmacopoeia (Ph. Eur.) 2.6.1. Briefly, a solution from a specified number of containers was filtered through a filter of nominal pore size 0.45 μm. Recovery of viable microorganisms from the filter(s) was performed by submerging the filter in fluid thioglycollate medium for anaerobic bacteria (at 32.5 ± 2.5 °C), and hydrolyzed soya-bean casein digest for fungi and aerobic bacteria (at 22.5 ± 2.5 °C), followed by incubation for 14 days.

#### 2.2.7. Efficacy of Antimicrobial Preservation

The efficacy of antimicrobial preservation was assessed according to the Ph. Eur. 5.1.3. [24]. Briefly, the formulations were challenged in their final containers, with a prescribed inoculum of *Pseudomonas aeruginosa* ATCC 9027, *Staphylococcus aureus* ATCC 6538, *Candida albicans* ATCC 10231 and *Aspergillus brasiliensis* ATCC 16404. The antimicrobial activity was expressed in terms of the log10 reduction in the number of viable microorganisms against the value obtained for the inoculum, applying the criteria for parenteral preparations.

#### 2.2.8. In Vitro Release Studies

The release of morphine hydrochloride from F1-MH and F2-MH gels was studied using Franz diffusion cells (3 mL receptor volume; 1 cm^2^ permeation area), provided with hydrophilic polysulfone membranes filters, using purified water as receptor phase, in *sink* conditions. The membranes were washed and equilibrated with the receptor phase during 24 h and then set between the donor and receiver compartments of the Franz diffusion cells. The donor phase consisted of 200 mg of each gel. The system was maintained at 32 ± 1 °C for about 30 min before the experiment started. The samples were then applied evenly on the surface of the membrane in the donor compartment and immediately sealed with Parafilm® to prevent water evaporation. Samples of the receptor phase (200 µL) were collected at 1, 2, 3, 4 and 6 h and the volume was replaced with fresh receptor phase kept at the same temperature. Studies were performed using six Franz cells per formulation and the amount of permeated drug was determined using the HPLC method described above.

The data obtained from in vitro release studies were computed using DDsolver [30], which is an Excel-plugin module, and the resultant data were fitted to different kinetic models [31]:

1) Zero order kinetics
  (1) $$F=K_{0}\times t$$
  Where, K_0_ is the zero order release constant
2) First order kinetics
  (2) $$F=100\times \left(1-e^{-K_{1}\;\times \;t}\right)$$
  Where, K_1_ is the first order release constant.
3) Higuchi model
  (3) $$F={\;K}_{H}\;\times \;t^{1/2}$$
  Where, K_H_ is the Higuchi release constant.
4) Korsmeyer-Peppas model
  (4) $$F=K_{KP}\times t^{n}$$
  Where, K_KP_ is the release constant incorporating the structural and geometric characteristics of the drug-dosage form, and n is the diffusional exponent indicating the drug-release mechanism.

In all models, F is the fraction (%) of released drug in time, t. The adjusted coefficient of determination (R^2^_adjusted_) was estimated for each model, fitted and used as a model ability to describe a given dataset. The R^2^_adjusted_ values and the Akaike minimum information theoretical criterion (AIC) were used as a measure of fit to compare the different models. When comparing several competing models, the best fitting model is that which gives the minimum AIC value [32]. The dissolution efficiency (DE) was also calculated from the area under the dissolution curve up to a certain time t, according to the following equation [33]:

(5) $$DE\;\left(\%\right)=\frac{\int_{0}^{t}y\times dt}{y_{100}\times t}\times 100$$

Where, y is the percentage of dissolved drug at time t. In the present study, DE was calculated at 6 h (DE_6h_).

#### 2.2.9. Stability Studies

Stability studies followed, where applicable, using the international guideline ICH Q1A(R2) [21]. Three batches of semi-solid and fluid gel formulations, packaged in the proposed container closure systems, were studied for 60 days at long-term (5 ± 3 °C), intermediate (22 ± 3 °C), and accelerated conditions (40 ± 2 °C/75 ± 5% Relative Humidity, using a Votsch Industrietechnik VC2033 climatic test chamber), all protected from light. Likewise, three batches of formulation F1-MH were studied for 60 days and exposed to light at 22 ± 3 °C. Samples were collected at 0, 7, 14, 30 and 60 days, and analysed for appearance, pH, viscosity, drug content and sterility (Ph. Eur. 2.6.1).

#### 2.2.10. Statistical analysis

Data were expressed as mean and standard deviation (mean ± SD) of separate experiments (n = 6). Statistical evaluation of data was performed using two-way analysis of variance (ANOVA). Differences were considered to be significant when p < 0.05.

## 3. Results and discussion

### 3.1. Risk Assessment

**Figure 2:**
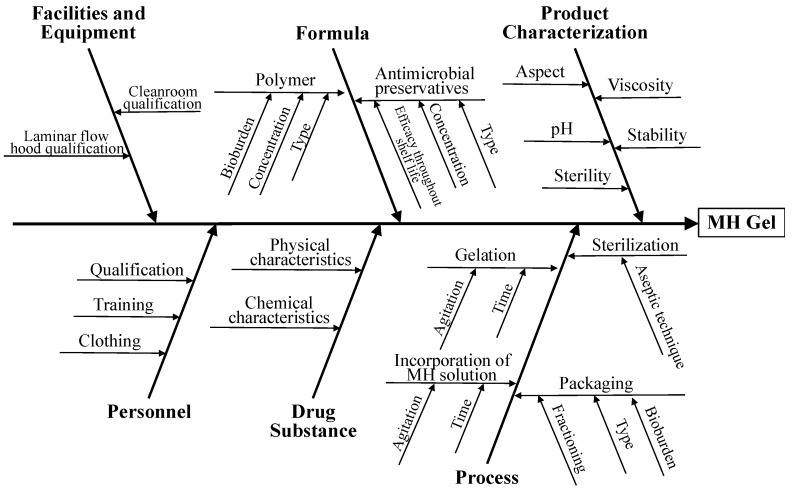
Ishikawa diagram illustrating factors that may have impact on the quality of MH-containing hydrogels for wound application.

The focus of the present work was the topical delivery of MH-containing hydrogels to painful wounds. The factors that potentially affect the quality attributes of the hydrogels were identified using an Ishikawa diagram (Figure 2). 

Concerning the polymer, the physicochemical characteristics of the commercially available semi-solid gels used in dressings and/or reported in the literature for similar applications were used as a reference (e.g. IntraSite® Gel). Thus, NaCMC was selected as a gel-forming agent, yielding gels for topical applications that facilitate cell rehydration, debridement and wound healing through early physical eradication of bacteria from the tissue bed surfaces [16]. NaCMC gels stimulate and accelerate wound-healing processes, probably because their negative charge promotes the binding to growth factors actively secreted to stimulate healing, similar to what occurs with the glycosaminoglycans present in cutaneous connective tissue [34]. The negatively charged NaCMC network may function as a reservoir of these molecules, which can be made available for epithelial migration [35].

**Figure 3:**
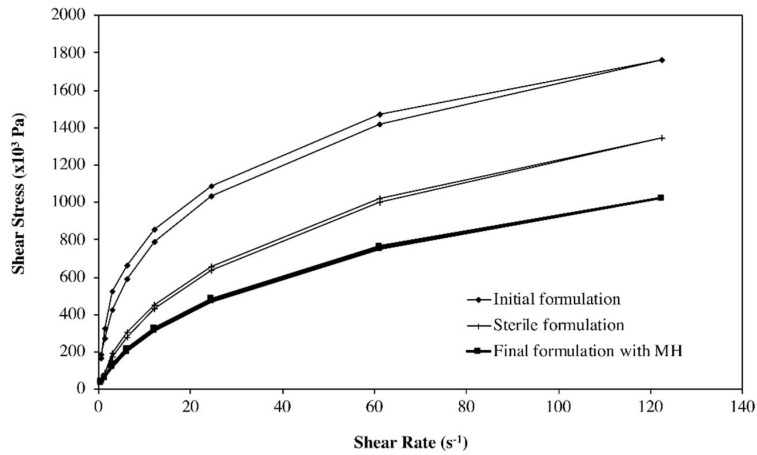
Flow curves of NaCMC gels (3%), before autoclaving (♦), after autoclaving (**+**) and after MH incorporation (■).

Studies were performed to evaluate the ability of the selected polymeric agent in maintaining a suitable rheological behavior throughout the different steps of the semi-solid and the fluid hydrogels’ preparation, particularly the influence of sterilisation by autoclaving at 121 °C/15 min. Aqueous solutions of NaCMC may be sterilised in an autoclave, although viscosity reductions of ca. 25% have been reported, depending on the molecular weight and degree of substitution [36]. For example, the apparent viscosity of the NaCMC semi-solid hydrogel (F1) was decreased (from 108 × 10^3^ Pa.s to 49.6 × 10^3^ Pa·s measured at 6.12 s^−1^) as a consequence of autoclaving, while maintaining its shear thinning behavior suitable for filling the syringes (final packaging) and for the intended topical application (Figure 3). These observations were confirmed for all formulations, during stability studies.

The pH is another important parameter for formulations intended for wound application. Both acute and chronic wounds usually present an alkaline pH, with values around 7.2 to 8.9. High alkaline pH values are associated with lower healing rates and, as healing occurs, there is a pH change from alkaline to a neutral and then acidic state [22]. Dressings that reduce the pH of wound fluid may help to prevent infection and produce conditions leading to more rapid healing than materials that are produced an alkaline local environments [22]. Likewise, NaCMC hydrogels present slightly acidic pH (6.37 ± 0.05 for the semi-solid F1 gel and 6.22 ± 0.04 for the fluid F2 gel), which are close to those of the commercial formulations described in the literature for similar applications (6.68 ± 0.05 for IntraSite® Gel and 5.83 ± 0.02 for Varihesive® Hydrogel). The acidic conditions help in wound healing by reducing infections, decreasing proteolytic activity, releasing oxygen and enhancing fibroblast proliferation and neovascularisation [37,38]. 

Antimicrobial preservation is another CQA of the formulation in which the pH of NaCMC hydrogels play an important role. Preservative selection must be based on a rational assessment of several interacting factors to optimise antimicrobial efficacy, such as pH, the nature of the dosage form, and the effect of containers. Several types of hydrocolloids decrease the antimicrobial activity of preservatives and NaCMC show some degree of incompatibility with quaternary ammonium compounds. In addition, most organic acids used as preservatives show no activity at pH > 6. Therefore, among the antimicrobial preservatives or combinations tested for the F1 and F2 gels, methylparaben and propylparaben, exhibiting activity at pH 4–8, were selected and proven to be efficient at the concentrations described in Table 2, according to the Ph. Eur. 5.1.3 [24].

Microbiological quality is a critical issue in pharmaceutical compounding [39]. The preparation technique was designed to ensure the quality and sterility of the final product. All compounding operations were performed in suitable facilities by qualified personnel, according to the USP <797> requirements [23]. As raw materials may be reservoirs of microorganisms and are considered one of the most important sources of drug products contamination, bioburden should be minimised throughout the manufacturing process [26]. High quality raw materials significantly reduce the risk of microbiological contamination of the final formulation, so careful selection was performed to assure supplier qualification, and the reliability of certificates of analysis.

Preparations intended for use on severely injured skin must be sterile not only to avoid healing process retardation, but also due to the fragility of the skin barrier function to pathogenic agents. Thus, gels were sterilised by autoclaving at 121 °C/15 min and any further processing was performed in aseptic conditions, under a controlled environment. As the degradation rate of MH increases with temperature [40], its incorporation in the F1 and F2 gels was performed only after the sterilisation step, using aseptic processing in a class A horizontal laminar flow hood, inside a class B cleanroom. Throughout filling and packaging, the handling of the container closure system and their sterility are crucial to maintaining the sterility of the product. Containers and closures were sterilised before filling in the horizontal laminar flow hood.

### 3.2. Characterisation

The aim of this study was to develop two improved reproducible hydrogel formulations containing MH, using suitable excipients and a simple methodology, feasible in the daily practice of compounding medicines in a hospital pharmacy. The active substance concentration was adapted according to the characteristics of the dose metering device used, however, the gap in clinical data on the use of preparations containing these features is still a limiting factor for the establishment of an optimal concentration hydrochloride morphine. A semi-solid gel containing 0.125% *w*/*w* of MH was developed using NaCMC and a commercially available MH solution for injection (Figure 1a and 1b). Also, a fluid gel containing 1.0% *w*/*w* of MH, intended for spraying, was developed by optimising the NaCMC concentration, allowing administration in wounds located in anatomic sites with difficult access or involving extensive areas (Figure 1c and 1d). The MH concentration in the semi-solid gel (0.125% *w*/*w*) was based on the literature as well as the current clinical practice [2,10]. Attending to the legal aspects related to MH commercial circuits, the fluid gel was prepared from a commercial injectable formula of MH 2% *w*/*w*, by incorporation in a equal volume of NaCMC 0.5% *w*/*w* to obtain the higher possible MH concentration, suitable for spraying in large anatomical zones and wounds where a consistent hydrogel is not an option. 

Odourless, yellowish, translucent and homogeneous hydrogel formulations were obtained (Figure 1a and c), with suitable acidic pH ≤ 6.4 (Table 3). A pH decrease from 6.22 ± 0.04 to 5.64 ± 0.01 was observed in the fluid gel after MH incorporation (Table 3), due to the pH of the MH injectable solution (4.8) and the high volume added (50 mL added to 50 g of F2 gel). Also suitable were the mean apparent viscosities of 50.1 × 10^3^ ± 3.9 × 10^3^ Pa·s (at 6.12 s^−1^ for F1-MH) and 20.6 ± 8.1 Pa·s (at 12.24 s^−1^ for F2-MH), facilitating easy gel application over painful wounds, particularly the latter, which is intended for spraying.

Although information about the metabolising capacity of human skin in the context of topically applied drugs and formulations is limited, evidence suggests that morphine biotransformation in the skin is minimal. Therefore, it should not be considered when designing a treatment regimen [6]. To simplify dose calculation, by mass or volume determination, density values were duly determined for F1-MH (1.021 g·cm^−3^) and F2-MH (1.010 g·cm^−3^).

### 3.3. In Vitro Release

**Figure 4:**
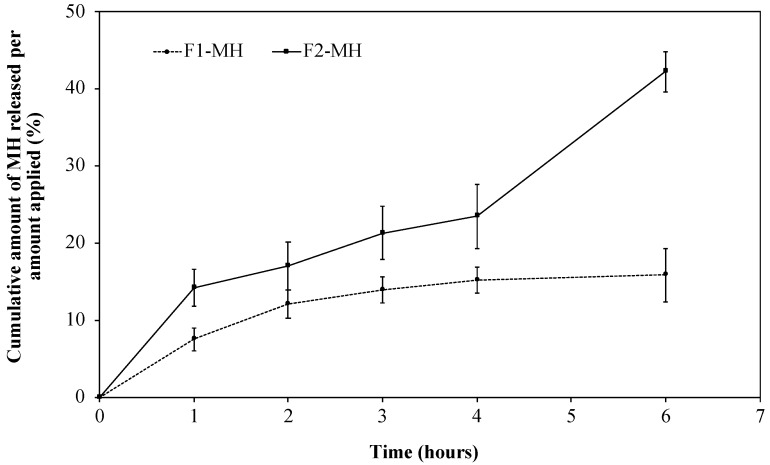
Release profiles of MH from the semi-solid hydrogel F1-MH (●) and the fluid hydrogel F2-MH (■), using vertical Franz diffusion cells (mean ± SD; *n* = 6).

The release of MH from the gel matrices was in general slow, allowing prolonged drug release with a reduction in the number of daily administrations (Figure 4). After 6 h, ca. 43.2% of the F1-MH drug content had been released, whereas the more fluid F2-MH formulation released 64.0% of the active substance, meeting the aims of each formulation. In the case of the semi-solid F1-MH formulation, intended for deeper wounds, the gel will stay in contact with the wound longer while releasing the active substance. The fluid F2-MH gel formulation, proposed for spraying over extensive wounds and/or surface or anatomic areas with difficult access, presents a higher release rate providing a rapid analgesic action. The difference in release rates between the two formulations is clearly associated to their different NaCMC concentrations and viscosities, which in turn are due the three-dimensional structures of the polymer network formed by the gelling agent in each formulation. Gels from hydrophilic polymers, such as cellulose derivatives, with higher viscosities, usually present higher resistance to dissolution and erosion and thus more sustained drug release profiles [41,42]. In the present study, the high concentration of thickening agent in the F1-MH formulation created a tighter polymeric network, thus decreasing the drug release rate as well as a lower DE_6h_ (12.7 ± 1.3%). In the case of the fluid gel (F2-MH), the low NaCMC concentration led to the formation of a looser polymeric network that facilitated MH release and increased DE6h (20.7 ± 3.6%). The final formulations exhibited release rates of 17.9 ± 2.2 μg·cm^−2^·h^−1^ (F1-MH) and 258.0 ± 30.4 μg·cm^−2^·h^−1^ (F2-MH).

For the characterisation of the MH release mechanism from the hydrogels, four different kinetic models were applied, i.e. zero order, first order, Higuchi and Korsmeyer–Peppas [31]. As the R^2^_adjusted_ coefficients are not sufficient on their own to compare the models, the AIC was also used as a measure of fit (Table 4).

Release data clearly do not fit the zero order or first order kinetics (Table 4). However, it should be noticed that the zero order kinetics has been observed for a MH-containing poloxamer 407 hydrogels, which has been attributed to the inclusion of glycerol and NaCMC in the formulation [18]. Instead, for the F1-MH hydrogel, the best fitting was obtained with the Higuchi square root model (R^2^_adjusted_ = 0.946 ± 0.023), with slightly lower values for the Korsmeyer-Peppas model (0.939 ± 0.041). The AIC values obtained for these models were not significantly different, which prevents a clear distinction between them (Table 4). Regarding the F2-MH, the Higuchi model also exhibits the best fitting (R^2^_adjusted_ = 0.923 ± 0.039) when compared to the Korsmeyer-Peppas model (0.923 ± 0.039), with close but distinct AIC values, with the Higuchi model presenting a clear minimum (Table 4). Data from both formulations appear to be consistent with a Fick’s diffusion process through the NaCMC polymeric matrix, which is the basis of the Higuchi’s model and is apparently confirmed by the n ≈ 0.5 release exponent obtained from the Korsmeyer-Peppas model (0.428 ± 0.008 for F1-MH and 0.480 ± 0.012 for F2-MH). As MH is dissolved in the NaCMC gel, this behavior is coherent with its homogeneous distribution in a uniform matrix, which acts as the diffusional medium.

### 3.4. Stability

During formulation development, preliminary package material compatibility and storage condition assays were carried out to find out the optimal conditions for the final formulations. Preliminary stability studies showed the NaCMC stock semi-solid (F1) and fluid (F2) hydrogels remained physically, chemically and microbiologically stable at room temperature during 60 days (data not shown). Similarly, both MH-containing formulations were physically, chemically and microbiologically stable at room temperature. Throughout the stability study, at the different tested temperatures the gels presented the same yellowish, transparent and homogeneous aspect, and MH concentrations remained within the 90–110% specification. At day 60, despite the temperature, the MH amount was ≥100% for all F1-MH and F2-MH batches tested, and no meaningful pH changes were observed in both gels, which presented values acceptable for application in wounds (Table 5). 

**Figure 5:**
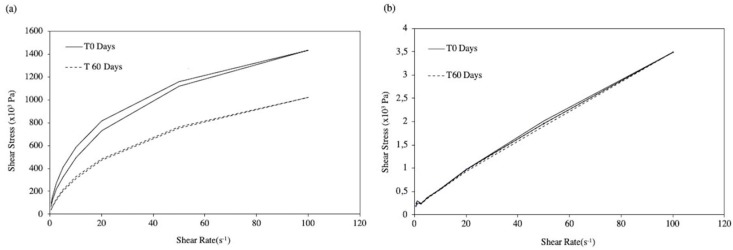
Flow curves of gel formulations F1-MH (**a**) and F2-MH (**b**) at room temperature along the stability study.

Overall these results confirm the data reported by Zeppetella and Ribeiro [15] using morphine sulfate in IntraSite® Gel. However, studies also revealed a general decrease in apparent viscosity, more pronounced as temperature increased, which is consistent with the reported NaCMC depolymerisation with prolonged heating [36]. Nevertheless, the variation in the apparent viscosity of F2-MH was not statistically significant for batches stored in the fridge or at room temperature (Table 5), which maintained their rheological behavior throughout the stability study, as depicted by the flow curves determined at room temperature (Figure 5b). 

Preservatives were effective (≥100% initial amounts for both gels, irrespective of storage conditions) and formulations complied with the Ph. Eur. requirements for the preservation efficacy for topical formulations, while remaining sterile throughout the study. Therefore, the formulations allowed establishing a beyond-use date of up to 60 days for the drug product stored at room temperature protected from light, which surpasses the initially proposed QTPP element (Table 1).

**Figure 6:**
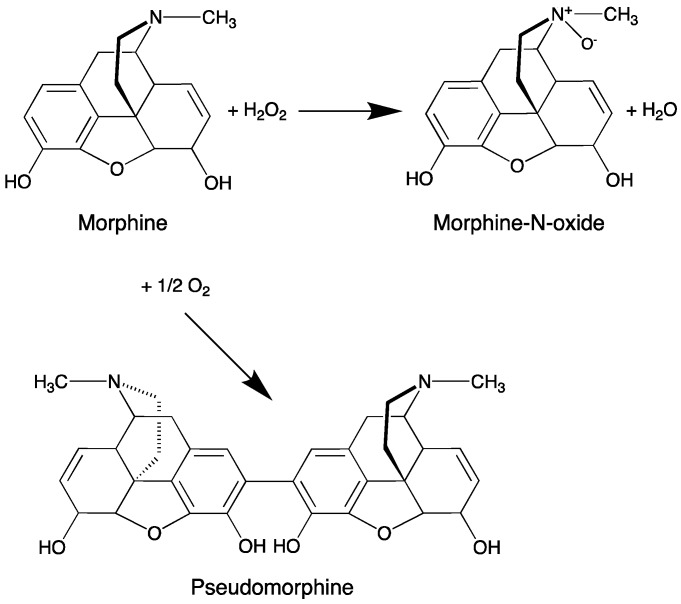
The most probable morphine degradation pathways, in aqueous solution under ambient conditions (adapted from [40]).

However, under light exposure, the semi-solid F1-MH gel stored showed a distinct discolouration to a dark yellow colour as well as ≈42% decrease in MH content (from 103.4 ± 2.4% at day 0 to 61.5 ± 3.4% at day 60). The degradation mechanism of morphine in aqueous solutions is well known, causing the discolouration of morphine solutions [43]. Oxygen, high pH values, sunlight, UV irradiation and metal ions may accelerate the degradation of morphine solutions and should be avoided when these are stored for long periods [37,41]. Morphine contains a phenolic hydroxyl group that degrades rapidly in neutral or alkaline solutions but is relatively stable in acidic conditions. Free radicals of morphine will likely be formed, causing dimerisation, thus generating pseudomorphine molecules, and also the formation of morphine-N-oxide (Figure 6). As the development of a yellow to brownish colour usually goes together with the formation of pseudomorphine and morphine N-oxide, it was also suggested that the discolouration is due to further degradation of these products [40]. It has been reported that when stored in syringes unprotected from light, morphine solutions showed up to 6-fold acceleration of the degradation when compared to the solutions stored under protection from light, presenting less than 50% of the initial concentration of morphine after 12 weeks [44]. Therefore, to avoid such degradation the F1-MH gel packed in unidose transparent syringes was further packed into a secondary packaging consisting of an opaque bag for light protection. For F2-MH this was not necessary, because the gel was packaged in amber Type II glass flasks that provided adequate protection against photodegradation. In fact, no discolouration or drug loss was detected in the F2-MH formulation stored at room temperature under light exposure (102.5 ± 0.1% at day 0 and 105.1 ± 0.5% at day 60).

## 4. Conclusions

In the absence of suitable options on the pharmaceutical market, compound formulations have been proposed for wound related pain relief in cancer palliative care or burn patients. Given the potential high impact of drug product quality on patient’s health, the risk associated to this type of drug products should be assessed and tightly controlled. The herein proposed semi-solid and fluid MH-containing hydrogel formulations presented suitable physicochemical, pharmaceutical and microbiological characteristics for topical application to painful wounds, while proving to be stable for up to 60 days at room temperature, protected from light. The active substance concentration was adapted according to the characteristics of the dose metering device and the slow release profile may allow reducing the number of applications.

## Figures and Tables

**Table 1:** Identification of quality target product profile (QTPP) of morphine hydrochloride (MH) gels.

QTPP Element	Target	Reference
Route of Administration	Topical in wounds	[9]
Strength	Semi-solid gel: 0.125% MH; Fluid gel: 1.0% MH	According to the characteristics of the dose metering device
Dosage Form	Hydrogel	[2]
Drug Product Critical Quality Attributes [19]	Aspect: colourless to yellowish transparent, homogeneous gel pH: slightly acidic: 5.5–6.5 Viscosity: Semi-solid gel: 50 ± 20 × 10^3^ Pa·s; Fluid gel: 25 ± 10 Pa·s Assay: 90–110% Antimicrobial preservation Sterility	[22] ≤ viscosity of commercial hydrogels (IntraSite® Gel Varihesive® Hydrogel) USP<795> [23] Ph. Eur. 5.1.3 [24] Ph. Eur. 2.6.1; USP<797> [23,24]
Stability	Beyond-use date ≥28 days at 5 ± 3 °C	[21]

**Table 2:** Composition of NaCMC semi-solid (F1) and fluid (F2) hydrogels.

Excipients	Concentration (% *w*/*w*)		Quality Standard	Pharmaceutical Function
	F1	F2		
NaCMC	3.0*	0.5 *	Ph. Eur.	Polymer
Glycerol	5.0	5.0	Ph. Eur.	Humectant
Metylparaben	0.10	0.10	Ph. Eur.	Preservative
Propylparaben	0.010	0.010	Ph. Eur.	Preservative
Water for injections	qs to 100	qs to 100	Ph. Eur.	Solvent

* Initial NaCMC gel concentrations. The semi-solid F1-MH gel is obtained by adding 5 mL of MH aqueous injectable solution to 80 g of F1 gel; the fluid F2-MH gel is obtained by 50 mL of MH aqueous injectable solution to 50 g of F2 gel.

**Table 3:** Main characteristics of the NaCMC semi-solid (F1-MH) and fluid (F2-MH) morphine-containing hydrogels (mean ± SD, n = 3).

Parameter	NaCMC Hydrogels		Formulations	
	F1	F2	F1-MH	F2-MH
Aspect	Colourless, transparent and homogeneous	Colourless, transparent and homogeneous	Yellowish, transparent and homogeneous	Slightly yellowish, transparent and homogeneous
MH (%)	-	-	0.125	1.0
pH	6.36 ± 0.02	6.22 ± 0.04	6.37 ± 0.05	5.64 ± 0.05
Viscosity (Pa s^−1^)	61.3 × 10^3^ ± 1.90 × 10^3^	113.3 ± 2.3	50.1 × 10^3^ ± 3.9 × 10^3^	20.6 ± 8.1

**Table 4:** Kinetic parameters obtained after fitting the release data from the formulations to different release models (mean ± SD, *n* = 6).

Formulation	Model	K	R^2^_adjusted_	AIC
F1-MH	Zero order	3.71 ± 0.36	0.619 ± 0.095	28.19 ± 1.77
	First order	0.04 ± 0.00	0.682 ± 0.084	27.07 ± 1.79
	Higuchi	7.84 ± 0.78	0.946 ± 0.023	15.78 ± 3.06
	Korsmeyer-Peppas	8.65 ± 1.06	0.939 ± 0.041	16.13 ± 4.39
		n - 0.428 ± 0.08		
F2-MH	Zero order	7.35 ± 0.24	0.878 ± 0.076	30.39 ± 2.89
	First order	0.09 ± 0.00	0.908 ± 0.063	28.78 ± 2.78
	Higuchi	14.33 ± 0.84	0.923 ± 0.039	27.39 ± 3.22
	Korsmeyer-Peppas	14.31 ± 1.57	0.916 ± 0.039	28.81 ± 11.04
		n - 0.48 ± 0.12		

K—release constant; R^2^_adjusted_—adjusted coefficient of determination; AIC—Akaike information criterion; n—release exponent.

**Table 5:** Physicochemical stability of semi-solid (F1-MH) and fluid (F2-MH) morphine-containing hydrogels stored protected from light, at different temperatures (mean ± SD, *n* = 3).

Time (days)	F1-MH			F2-MH		
	Recovery of morphine (%)	pH	Viscosity (× 10^3^ Pa·s)	Recovery of morphine (%)	pH	Viscosity (Pa·s)
**Batches stored in the fridge (5 ± 3 °C)**						
0	100.4 ± 1.3	6.37 ± 0.05	50.1 ± 3.9	102.5 ± 0.1	5.64 ± 0.05	20.6 ± 8.1
7	101.7 ± 0.3	6.38 ± 0.07	47.7 ± 3.4	101.9 ± 0.1	5.59 ± 0.03	22.2 ± 9.4
14	104.0 ± 1.2	6.28 ± 0.03	47.1 ± 3.0	100.9 ± 0.9	5.64 ± 0.03	16.1 ± 3.3
30	104.9 ± 3.2	6.38 ± 0.04	48.2 ± 3.2	102.1 ± 0.2	5.67 ± 0.06	16.1 ± 4.2
60	104.7 ± 0.1	6.59 ± 0.03	41.2 ± 1.6	102.6 ± 1.6	5.61 ± 0.04	17.2 ± 2.6
**Batches stored at room temperature (22 ± 3 °C)**						
0	100.4 ± 1.3	6.37 ± 0.05	50.1 ± 3.9	102.5 ± 0.1	5.64 ± 0.05	20.6 ± 8.1
7	105.0 ± 0.7	6.32 ± 0.04	46.7 ± 4.7	101.6 ± 0.9	5.62 ± 0.11	25.0 ± 6.6
14	108.7 ± 4.3	6.37 ± 0.09	45.0 ± 3.7	101.0 ± 0.5	5.62 ± 0.02	16.7 ± 3.5
30	106.4 ± 0.2	6.39 ± 0.05	46.8 ± 6.7	101.3 ± 0.7	5.68 ± 0.05	17.8 ± 3.6
60	102.6 ± 2.7	6.57 ± 0.03	33.6 ± 2.0	103.6 ± 0.6	5.51 ± 0.03	17.8 ± 5.7
**Batches stored at accelerated conditions (40 ± 2 °C/75% Relative Humidity)**						
0	100.4 ± 1.3	6.37 ± 0.05	50.1 ± 3.9	102.5 ± 0.1	5.64 ± 0.05	20.6 ± 8.1
7	104.1 ± 1.9	6.30 ± 0.03	38.1 ± 2.5	101.0 ± 1.1	5.54 ± 0.03	17.2 ± 4.4
14	101.3 ± 2.0	6.29 ± 0.12	32.4 ± 1.4	102.7 ± 1.3	5.55 ± 0.02	22.2 ± 5.7
30	102.0 ± 0.8	6.34 ± 0.06	30.0 ± 1.7	104.0 ± 1.9	5.50 ± 0.04	15.0 ± 4.3
60	101.6 ± 1.3	6.39 ± 0.05	11.3 ± 0.6	105.5 ± 1.5	5.33 ± 0.03	11.1 ± 2.2
